# Complete mitochondrial genome of a woolly mammoth (*Mammuthus primigenius*) from Maly Lyakhovsky Island (New Siberian Islands, Russia) and its phylogenetic assessment

**DOI:** 10.1080/23802359.2018.1473721

**Published:** 2018-05-18

**Authors:** Igor V. Kornienko, Tatiana G. Faleeva, Natalia V. Oreshkova, Semyon E. Grigoriev, Lena V. Grigoreva, Evgeniy P. Simonov, Anna I. Kolesnikova, Yuliya A. Putintseva, Konstantin V. Krutovsky

**Affiliations:** a Department of Strategic Research, Southern Scientific Centre, Russian Academy of Sciences, Rostov-on-Don, Russian Federation;; b Laboratory of Biological Objects Identification, Southern Federal University, Rostov-on-Don, Russian Federation;; c Department of Forensic Medicine, Mechnikov North-Western State Medical University, Russian Federation;; d Laboratory of Forest Genetics and Selection, V. N. Sukachev Institute of Forest, Siberian Branch of Russian Academy of Sciences, Krasnoyarsk, Russian Federation;; e Laboratory of Forest Genomics, Genome Research and Education Center, Siberian Federal University, Krasnoyarsk, Russian Federation;; f Institute of Applied Ecology of the North, North-Eastern Federal University, Yakutsk, Russian Federation;; g Department of Forest Genetics and Forest Tree Breeding, Georg-August University of Göttingen, Göttingen, Germany;; h Laboratory of Population Genetics, Vavilov Institute of General Genetics, Russian Academy of Sciences, Moscow, Russian Federation;; i Department of Ecosystem Science and Management, Texas A&M University, College Station, TX, USA

**Keywords:** Ancient DNA, elephantidae, North-Eastern Siberia, mitogenome, Maly Lyakhovsky island

## Abstract

We present a complete sequence and an annotation of the mitochondrial genome of the woolly mammoth (*Mammuthus primigenius*) found in 2012 on Maly Lyakhovsky Island (North-Eastern Siberia, Russia). The genome was 16,851 bp long and contained 13 protein-coding, 22 tRNA, and 2 rRNA genes. It was AT reach (61.3%) with A = 32.9%, T = 28.4%, C = 25.3%, and G = 13.4%.

A partial carcass of a woolly mammoth female adult (*Mammuthus primigenius*) was found on the Maly Lyakhovsky Island (74°13'N, 141°03'E) of the New Siberian Islands archipelago in 2012. The remains contained relatively well-preserved soft tissues in some parts of the body. The anatomy, morphology, haematology, histology, and composition of intestinal microbial communities of this specimen were described in Grigoriev et al. (2017). To examine the level of DNA integrity in the specimen, and to study its phylogenetic relationships with other woolly mammoths, the tissue samples were collected for DNA extraction and sequencing of complete mitochondrial genome. The specimen is stored in the Mammoth Museum of North-Eastern Federal University (number 1589).

Samples of trunk muscle tissue were taken with sterile scalpel and scissors. Bone fragments were taken from a brachial bone and a rib. DNA isolation from each sample was performed using the PrepFiler BTA Forensic DNA Extraction Kit (Applied Biosystems/Thermo Fisher Scientific Co., Waltham, MA), DNA IQ System (Promega Corporation, Madison, WI) and phenol–chloroform purification with modifications. An appropriate negative control was used at every step.

PCRs were carried out in GeneAmp PCR System 9700 (Applied Biosystems, Foster City, CA) using a set of primers covering whole mitochondrial genome and specifically designed for this study using Primer3 program (Untergasser et al. 2012). Sequencing was performed on an ABI 3130XL automatic sequencer (Applied Biosystems) using the Big-Dye®Terminator 3.1 kit (Applied Biosystems) and the same primers that were used for PCR amplification. The detailed DNA extraction protocol, amplification conditions, and primer sequences are provided in supplementary materials (available at https://doi.org/10.6084/m9.figshare.6157850).

All available complete and partial mitochondrial genomes of other mammoths were downloaded from GenBank, aligned using Clustal Omega (Sievers et al. 2011) and trimmed manually for repetitive regions and gaps. The phylogenetic trees were generated using the maximum likelihood (ML) method and IQ-TREE 1.6.3 (Nguyen et al. 2015) with 1000 ultrafast bootstrap replicates.

We were able to amplify the fragments up to 1500 bp long (Figure S2 in Supplementary material) confirming a relatively good DNA preservation considering the age of the specimen (32480–32930 years; Grigoriev et al. 2017). To the best of our knowledge, the only study, where similar level of amplification efficiency was achieved for a DNA, was performed on genomic DNA extracted also from the muscle tissue of a mammoth (Rogaev et al. 2006).

The mitogenome of our *M. primigenius* specimen was 16,851 bp long (NCBI GenBank accession number MF770243) and based on annotation included 13 protein-coding, 22 tRNA, and 2 rRNA genes in a typical vertebrate gene order. Short tandem repeats (ACGCAT)_≥40_ were found between conserved sequence block 1 (CSB1) and CSB2 of the control region. It is difficult to estimate certain number of repeats due to possible polymerase stuttering or real heteroplasmy (Rogaev et al. 2006).

Woolly mammoths belonging to the two major phylogenetic lineages are known to occur in North-Eastern Siberia (Chang et al. 2017). The studied specimen was clustered together with the members of ‘Clade 1’ (as defined in Chang et al. 2017; Figure 1).

**Figure 1.**
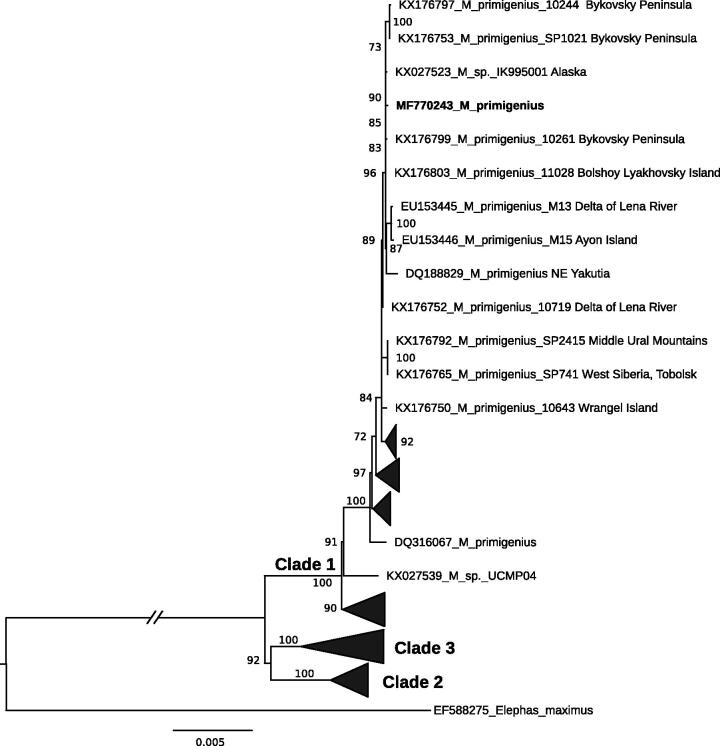
Maximum likelihood phylogenetic tree of *Mammuthus primigenius* based on 16,444 bp long alignment of 137 publically available mitogenomes including the studied specimen from Maly Lyakhovsky Island highlighted in bold font. Asian elephant (*Elephas maximus*) was used as an out-group. The best-fit substitution model TN93 + F + R3 averaged for a whole mitogenomes alignment was chosen according to Bayesian information criterion among 286 tested models in ModelFinder (Kalyaanamoorthy et al. 2017). GenBank accession numbers and ultrafast bootstrap values above 70 are presented in the tree.

## Supplementary Material

Supplemental Material

## References

[CIT0001] Chang D, Knapp M, Enk J, Lippold S, Kircher M, Lister A, MacPhee R, Widga C, Czechowski P, Sommer R, et al 2017 The evolutionary and phylogeographic history of woolly mammoths: a comprehensive mitogenomic analysis. Sci Rep. 7:1–10. 28327635 10.1038/srep44585 PMC5361112

[CIT0002] Grigoriev SE, Fisher DC, Obadă T, Shirley EA, Rountrey AN, Savvinov GN, Garmaeva DK, Novgorodov GP, Cheprasov MY, Vasilev SE. 2017 A woolly mammoth (*Mammuthus primigenius*) carcass from Maly Lyakhovsky Island (New Siberian Islands, Russian Federation). Quat Int. 445:89–103.

[CIT0003] Kalyaanamoorthy S, Minh BQ, Wong TFK, von Haeseler A, Jermiin LS. 2017 ModelFinder: fast model selection for accurate phylogenetic estimates. Nat Methods. 14:587–589. 28481363 10.1038/nmeth.4285 PMC5453245

[CIT0004] Nguyen LT, Schmidt HA, von Haeseler A, Minh BQ. 2015 IQ-TREE: a fast and effective stochastic algorithm for estimating maximum likelihood phylogenies. Mol Biol Evol. 32:268–274. 25371430 10.1093/molbev/msu300 PMC4271533

[CIT0005] Rogaev EI, Moliaka YK, Malyarchuk BA, Kondrashov FA, Derenko MV, Chumakov I, Grigorenko AP. 2006 Complete mitochondrial genome and phylogeny of Pleistocene mammoth *Mammuthus primigenius*. PLoS Biol. 4:e73. 16448217 10.1371/journal.pbio.0040073 PMC1360101

[CIT0006] Sievers F, Wilm A, Dineen D, Gibson TJ, Karplus K, Li W, Lopez R, McWilliam H, Remmert M, Söding J, et al 2011 Fast, scalable generation of high-quality protein multiple sequence alignments using Clustal Omega. Mol Syst Biol. 7:539. 21988835 10.1038/msb.2011.75 PMC3261699

[CIT0007] Untergasser A, Cutcutache I, Koressaar T, Ye J, Faircloth BC, Remm M, Rozen SG. 2012 Primer3 – new capabilities and interfaces. Nucleic Acids Res. 40:e115. 22730293 10.1093/nar/gks596 PMC3424584

