# Supplementary material for: Complete mitochondrial genome of a woolly mammoth (Mammuthus primigenius) from Maly Lyakhovsky Island (New Siberian Islands, Russia) and its phylogenetic assessment
Source: Mitochondrial DNA B Resour. 2018 May 18;3(2):596–8. doi: 10.1080/23802359.2018.1473721 (PMC7800944; doi:10.1080/23802359.2018.1473721)
Supplement: Supplemental Material [file TMDN_A_1473721_SM5250.pdf]

SUPPLEMENTARY MATERIAL

Sample preparation, DNA isolation and amplification protocol used in **“Complete mitochondrial genome of a woolly mammoth (*Mammuthus primigenius*) from Maly Lyakhovsky Island (New Siberian Islands, Russia) and its phylogenetic assessment”** Igor V. Kornienko, Tatiana G. Faleeva, Nataliay V. Oreshkova, Semyon E. Grigoriev, Lena V. Grigoreva, Evgeniy P. Simonov, Anna I. Kolesnikova, Yuliya A. Putintseva and Konstantin V. Krutovsky

Sample preparation before DNA isolation

Bones

The bone fragments were placed in separate containers and washed with tap water for 10 minutes, then the upper layer of compact bone was removed by a portable dental drill and small bone fragments (50 × 50 mm) were obtained with help of oscillating saw. These bone fragments were cleaned with "Alaminol" washing solution diluted 1:10 with tap water, followed by treatment with 7 mM NaOCl. Each bone fragment was then washed with sterile deionized water. At the next step the fragments were placed into sterile Petri dishes and dried under ultraviolet radiation (wavelength 254 nm, 40 W) for 15 min from each side of the bone. Then the bone fragments were left in open Petri dishes for one day at room temperature to dry completely. Retsch MM200 Mixer Mill was used to grinding the bone samples into homogenized powder. The bone powders were transferred to separate sterile 15 ml tubes and stored at -70 ° C.

Muscle tissue

Fragments of muscle tissue about 5 × 5 mm in size were cut from the deep layers of soft tissues of the trunk using a sterile scalpel and scissors. They were placed into sterile 2 ml tubes and cut into small fragments.

DNA isolation

We used the following DNA extraction protocols and methods:

1. PrepFiler BTA Forensic DNA Extraction Kit (Applied Biosystems) according to manufacturer’s instructions.
2. DNA IQ System (Promega) according to manufacturer’s instructions.
3. Phenol-chloroform extraction with modifications (see below).

About 150 mg of muscle tissue were placed into 1.5 ml tubes with 550 μl lysing buffer (10 mM Tris-HCl, pH 7.5; 10 mM Na_2EDTA; 50 mM NaCl; 2% SDS) with 15 μl proteinase K (20 mg/ml), and 10 μl of 1 M DDT. For decalcification of bone powder, 10 ml 0.5 M EDTA were added to 0.5 g of the bone powder, mixed thoroughly for 1 min, centrifuged at 10,000 g for 3 min, and discarded supernatant. Then, 4.5 ml of lysing buffer (10 mM Tris-HCl, pH 7.5; 10 mM Na_2EDTA; 50 mM NaCl; 2% SDS) with 200 μl of proteinase K (20 mg/ml) and 200 μl of 1 M DDT were added.

All samples were mixed thoroughly and incubated at +56 ˚C for one hour, and at +37 ˚C for 24 hours. Then samples were centrifuged for 5 min at 12,000 g, and supernatant was transferred to new sterile tubes, followed by addition of equal volume of phenol/chloroform/isoamyl alcohol mix (25:24:1) and vortexed. This mixture was centrifuged for 5 min at 12000 g, and upper water phase was transferred to new tubes. The phenol/chloroform/isoamyl alcohol extraction was repeated twice. At the following step, an equal volume of n-butanol was added to the water solution of DNA, vortexed shortly, and centrifuged for 5 min at 12000 g. Upper phase of n-butanol was removed, leaving water solution of DNA, which was cleaned and concentrated by AmiconUltra-4 30K centrifugal filter units. The final volume of all DNA extracts was 50 μl.

The negative control was used with every DNA extraction method.

PCR and sequencing

Amplification of DNA was carried out in GeneAmp PCR System 9700 (Applied Biosystems, USA) using a set of primers covering whole mitochondrial genome and specifically designed for this study (Table S1). PCR was performed in a 50 µl volume each containing 5 µl DNA, 5 µl of 10× amplification buffer, 5 µl of 25 mM $MgCl_2$, 4 µl of 2.5 mM dNTP mix, 2 µl of BSA (3.2 mg/ml), 2.5 U of AmpliTaq DNA Polymerase (Applied Biosystems), and 2 µl of each primer (1 OD). An initial denaturation step was set for 2 min at 95°C, followed by 35 cycles of 30 s at 95°C, annealing at primer-specific temperature (see Table S1) for 20 s, 2.5 min extension at 72°C, and a final extension of 10 min at 72°C. The PCR products were cleaned with GeneJET PCR Purification spin columns (Thermo Fisher Scientific). Sequencing was performed on an ABI 3130XL automatic sequencer (Applied Biosystems / Thermo Fisher Scientific) using the Big-Dye®Terminator 3.1 kit (Applied Biosystems / Thermo Fisher Scientific) and the same primers that were used for amplification.

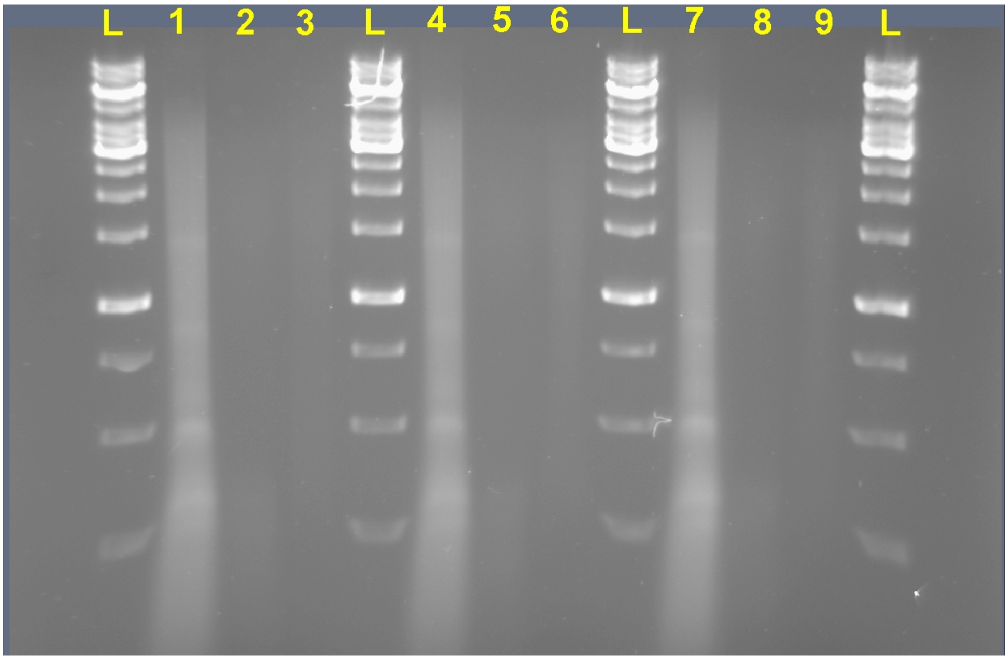

Figure S1. Agarose gel electrophoresis (1%) of total DNA extracted from trunk muscle tissue of woolly mammoth by different methods: phenol/chloroform (1, 4, 7), PrepFiler BTA Forensic DNA Extraction Kit (2, 5, 8), DNA IQ System (3, 6, 9). GeneRuler 1 kb Plus DNA Ladder (Thermo Fisher Scientific, Waltham, MA, USA) was used as size standard (L).

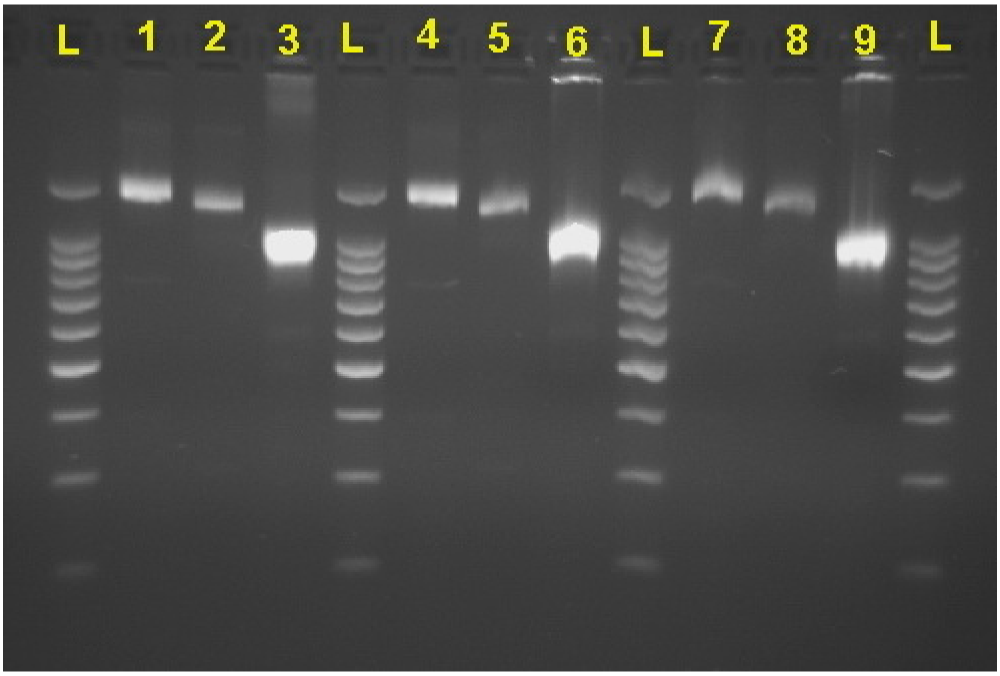

Figure S2. Agarose gel electrophoresis (2%) of PCR products obtained using DNA of woolly mammoth: 1, 4, 7 – 1474 bp long fragment amplified using the PCR primers 15377F and 16789R; 2, 5, 8 – 1338 bp long fragment amplified using the PCR primers 15513F and 16789R); 3, 6, 9 – 988 bp long fragment amplified using the PCR primers 15863F and 16789R); L – 100 bp DNA ladder.

Table S1. Primers used to amplify and sequence mitochondrial genome of *Mammuthus primigenius* from Maly Lyakhovsky island

Pair number	Primer name*	Primer sequence (5'-3')	T_A, ˚C
1	327F<br>1214R	GGCCATACGATTAGTCCAAA<br>TTTCATCATTCCCTTGCGGTA	53
2	1101F<br>1900R	CTAAATCTAGCCCTACCAACC<br>TTTATGTTTGCCGAGTTCC	53
3	1589F<br>2521R	ATTAATTTCATAGTTGGCTTGG<br>ACTATAGATAGAAACCGACCTG	53
4	2344F<br>3322R	TCAACGGAACAAGTTACCCTA<br>TTAAATCAAATGGAGCTCGG	54
5	3108F<br>3981R	CAAATTCAAAATACGCCCTC<br>TCCTATTCAGGCTGTTAGTCA	53
6	3934F<br>5084R	TAACAACACTACTCGCAGGA<br>AGCCCTCCAATCTAGAAGG	53
7	5029F<br>6271R	AAGATATCACCTTACATCAACTG<br>GACTTTTACGCCAGTTGGAA	53
8	5328F<br>6271R	CCTATGTTTGCTAACCGCTGA<br>GACTTTTACGCCAGTTGGAA	54
9	6154F<br>6997R	GGGCTATAATATCAATTGGCT<br>AATTTACGGGATTTAAACCTG	53
10	6810F<br>7599R	TGACTCAACGGATGTCCAC<br>CAGATTTCGGAACATTGACCA	53
11	7335F<br>8216R	CTGGAGCTATGAATATACTGA<br>TTGCTATGCTTAAGTTTACGGTT	52
12	8021F<br>8821R	TCCAAATCGCCTAATTACCAAC<br>GTAAACCTTCTTGCACGACA	53
13	8672F<br>9651R	GCCCTACTTATAACATCTGG<br>AGTAATGCGATTTCTAGGTCA	53
14	9314F<br>10128R	GCTGCTTGATATTGACATTTCG<br>TTTTGTACGTAGTCTAGTCCG	52
15	10020F<br>10991R	CAATATAAACTTCACGTTGTCT<br>TTCATATCTGTTTGTCGCAAG	53
16	10834F<br>11719R	CAATCGCTGGATCTATAGTGT<br>ACTTTTATCTGGAGTTGCAC	53
17	11442F<br>12263R	AAACCTTCATTTACCCGAGA<br>GCCTATATCACCGATACGAT	53
18	12137F<br>13104R	GCGCTAATAACCTACTACAAC<br>TGCTGCCTAGGATTAATCGT	54
19	12997F<br>13846R	GCAAGTTACAGTACCCGAA<br>ATACTATTGCTATGGCTACTGA	53

Pair number	Primer name*	Primer sequence (5’-3’)	T_A, °C
20	13797F<br>14750R	AAATGCACTCAAAACTACAAC<br>TTATTTGAGCCTGTTTCGTGA	53
21	14468F<br>15313R	CTACCTATACTCGGAAACCTG<br>TCATTTATGGCTTACAAGACC	53
22	15069F<br>16006R	CTACATACATCTAAACACCGAA<br>CGGCCATAGCTGAATCACA	53
23	15377F<br>15916R	CGCTATCAATACCCAAAACTGA<br>CTTATTTAAGAGGAAAGAG	55
24	15513F<br>16378R	GACCATACTATGTATAATC<br>CGTATGCGTATGCGTATAC	55
25	15863F<br>16789R	TCTTACTTCAGGACCATCT<br>CAAATCTAGGAACATGGCTCTT	55
26	15377F<br>16789R	CGCTATCAATACCCAAAACTGA<br>CAAATCTAGGAACATGGCTCTT	55
27	15513F<br>16789R	GACCATACTATGTATAATC<br>CAAATCTAGGAACATGGCTCTT	55
28	29F<br>629R	TACTGAAAATACCTAGACGAG<br>GCTTTACGCCGTATGCTTA	54
29	133F<br>629R	TGAAATCAAGAGTATAAACGGGAG<br>GCTTTACGCCGTATGCTTA	55
30	29F<br>541R	TACTGAAAATACCTAGACGAG<br>ACTACTGCTGTTTCCCGTG	55
31	1950F<br>2477R	AGCATTACTAGTATTCGAGGCA<br>CACGTAGGACTTTAATCGTTG	54
32	3663F<br>4511R	TTGATTTCACTAGCATGCATCCCA<br>AAATGTAAATGGCTAAGTTCAGGA	54
33	4600F<br>5481R	CTAATATGAAACAAAACACCT<br>AATAACATTATAGATTTGGTCG	52
34	4600F<br>5554R	CTAATATGAAACAAAACACCT<br>GTTTCCAAAGCCTCCAAT	52
35	11917F<br>12806R	AGAAGCCTATATCTCTAACTGAC<br>GTCCTCCTATTTTGCGGAT	54
36	13619F<br>14033R	AATCAAAACCCATAGCTATACAAA<br>TATTATGAGTGTGCTTTATGTGGT	53
37	16591F<br>50R	AGTTACCCTTGGCTCAATTTC<br>ACTCGTCTAGGTATTTTCAG	58
38	16591F<br>60R	AGTTACCCTTGGCTCAATTTC<br>AGTTGGATATACTCGTCTAGG	55
39	16550F<br>50R	GAAGAGATAGTTACATAGTGG<br>ACTCGTCTAGGTATTTTCAG	58

Pair number	Primer name*	Primer sequence (5'-3')	T_A, ˚C
40	16550F	GAAGAGATAGTTACATAGTGG	58
	60R	AGTTGGATATACTCGTCTAGG	
41	16142F	AACCTAAATTCCCAGCGTAC	58
	16789R	CAAATCTAGGAACATGGCTCTT	
42	16146F	TAAATTCCCAGCGTACACGC	58
	16789R	CAAATCTAGGAACATGGCTCTT	
43	16142F	AACCTAAATTCCCAGCGTAC	58
	16611R	GAAATTGAGCCAAGGGTAAC	

* The numbers in the primer name represent the corresponding nucleotide positions in the reference mitogenome used to design the primers (Genbank accession number DQ316067).
